# Isolation and molecular characterization of lumpy skin disease virus from hard ticks, *Rhipicephalus* (*Boophilus*) *annulatus* in Egypt

**DOI:** 10.1186/s12917-022-03398-y

**Published:** 2022-08-05

**Authors:** Ramy E. El-Ansary, Wahid H. El-Dabae, Ahmed S. Bream, Abeer El Wakil

**Affiliations:** 1 Zoology and Entomology Department, Faculty of Science Al-Azhar University, Cairo, Egypt; 2 Microbiology and Immunology Department, Veterinary Research Division, National Research Centre, Giza, 12622 Dokki Egypt; 3 Biological and Geological Sciences Department, Faculty of Education, Alexandria University, Alexandria, Egypt

**Keywords:** Lumpy skin disease virus, *Rhipicephalus annulatus*, Ticks, Egypt, Cattle disease

## Abstract

**Background:**

Lumpy skin disease (LSD), a disease of cattle and buffaloes, has recently become widely prevalent in Egypt. The aim of this study was to ascertain the potential role of *Rhipicephalus (Boophilus) annulatus* ticks in the transmission of this disease. Samples collected from suspected lumpy skin disease virus (LSDV) infected cows that had previously been vaccinated with the Romanian sheep pox virus (SPPV) in various Egyptian governorates were obtained between May to November over two consecutive years, namely 2018 and 2019. Ticks were morphologically identified and the partial cytochrome oxidase subunit I gene (COI) were sequenced, revealing that they were closely related to *R. (Boophilus) annulatus. *The G-protein-coupled chemokine receptor (GPCR) gene of the LSDV was used to test hard ticks.

**Results:**

Two positive samples from Kafr El-Sheikh province and one positive sample from Al-Behera province were reported. BLAST analysis revealed that the positive samples were closely related to the Kazakhstani Kubash/KAZ/16 strain (accession number MN642592). Phylogenetic analysis revealed that the GPCR gene of the LSDV recently circulating in Egypt belongs to a global cluster of field LSDV with a nucleotide identity of 98–100%. LSDV isolation was successfully performed four days after inoculation using 9 to 11-day-old embryonated chicken eggs showing characteristic focal white pock lesions dispersed on the choriallantoic membrane after three blind passages. Intracytoplasmic inclusion bodies, cell rupture, vacuoles in cells, and virus particles ovoid in shape were demonstrated by electron microscopy.

**Conclusion:**

In this study the role of hard ticks in the transmission of the LSDV to susceptible animals in Egypt was revealed and confirmed by various methods.

## Background

Ticks are the most common ectoparasites of livestock in tropical and subtropical regions causing significant economic losses due to their ability to transmit protozoan, rickettsial, and viral diseases [1]. They feed on the blood of mammals, birds, and occasionally reptiles and amphibians. Ticks were the first arthropods to be identified as pathogen vectors, and they are recognized as the primary arthropod vectors of disease agents to humans and domestic animals worldwide, alongside mosquitoes [2, 3]. The pathogen develops and multiplies in the vector, then spreads to humans and animals via the bite or excreta of arthropods such as mosquitos, tsetse flies, body lice, fleas, and ticks [4]. Among the major health and management issues for livestock, in many developing countries, are tick-borne protozoan diseases such as babesiosis and theileriosis, and rickettsial diseases such as anaplasmosis, cowdriosis, and tick-associated dermatophilosis. The genera *Hyalomma, Boophilus, Rhipicephalus*, and *Amblyomma* are the most economically important ixodid ticks in tropical areas [5].

LSDV, which belongs to the genus *Capripoxvirus* (*CaPV*), is a member of the family poxviridae with characteristic poxvirus  morphology and is closely related to sheep and goat pox viruses [6]. LSDV was first isolated in Egypt from infected cattle imported in 1988–1989 from Somalia causing two outbreaks in the governorates of Suez and Ismailya [7]. Bovine herpesvirus-4 (BHV-4) and LSDV were identified in a pooled sample from the initial outbreak (Suez). It was detected in 22 of 26 Egyptian governorates during the summer of 1989, with a low morbidity rate (2%) of the entire cattle population due to the rapid response of veterinary services, which resulted in the vaccination of nearly two million cattle with a live attenuated Romanian sheep pox vaccine. In 1998, an outbreak of LSD was reported in cattle in Upper Egypt's Menia Governorate [8]. This was followed by severe cyclical LSD outbreaks in 2006, 2011, 2012, and 2013 in cattle of several Egyptian governorates including Beni Suef, Al-Behera, Ismailya, Qalyubia, Dakahlia, and New Valley have been reported [9–14]. The phylogenetic analysis revealed that the Egyptian isolates from the 2006 outbreak were identical to LSDV [15]. Notifications and follow-up reports from the World Organization for Animal Health (OIE) indicated that an LSD outbreak occurred in Egypt in 2014, with the source suspected to be illegal animal movement or vectors [16]. The outbreak was suspected to have occurred in the Sharqiya governorate between January 2014 and May 2015 among cows and buffalos [17]. Since then, several cases have been reported across Egypt as a result of infected cattle imported from Ethiopia to the private quarantine at the Ismailya governorate, Egypt. LSD has been prevalent in different Egyptian governorates including Menofya, ElQalyubia, Kafr El-Sheikh, Al-Behera, Assuit, and Demiatta, with increased severity, causing severe losses in cattle, raising concerns that the disease continued to spread despite extensive vaccination campaigns, as reported in previous studies [18, 19].

Currently, it is widely suspected that LSDV is mechanically transmitted via arthropod vectors including mosquitos like the female *Aedes aegypti*, *Anopheles stephensi*, and *Culex quinquefasciatus*, as well as the biting midge *Culicoides nubeculosus* [20]. Recent molecular evidences of *R. (Boophilus) decoloratus* ticks transmitting LSDV transstadially and transovarially, as well as mechanical transmission by male ticks [21] or intrastadial transmission by *R. appendiculatus* and *Amblyomma hebraeum* ticks [22] have been reported. Moreover, transstadial transmission by *A. hebraeum* adults, moulted from nymphs fed on experimentally infected cattle, has been shown [23]. LSDV has been detected in eggs [22] and larvae hatched from eggs laid originating from *A. hebraeum* and *R. (Boophilus) decoloratus* females previously fed on infected cattle also tested positive by real-time PCR and virus isolation [24].

The novel vaccine policy all over the world depends on preparation of homologous genotype matched vaccine against circulating field strain to improve protection level, minimize post  vaccination reaction and decrease virus shedding. Also, the determination of the circulating field virus helps us to ameliorate the vaccination programs. Therefore, the present study aimed to investigate and validate recent LSDV outbreaks isolated from ticks collected from various governorates in Egypt and characterize the virus at the molecular level in various regions of Egypt. Additionally, LSDV were inoculated into the CAM of SPF-ECEs and transmission electron microscopy (TEM) analysis was performed to confirm viral infection.

## Results

### Tick collection and species identification

**Fig. 1:**
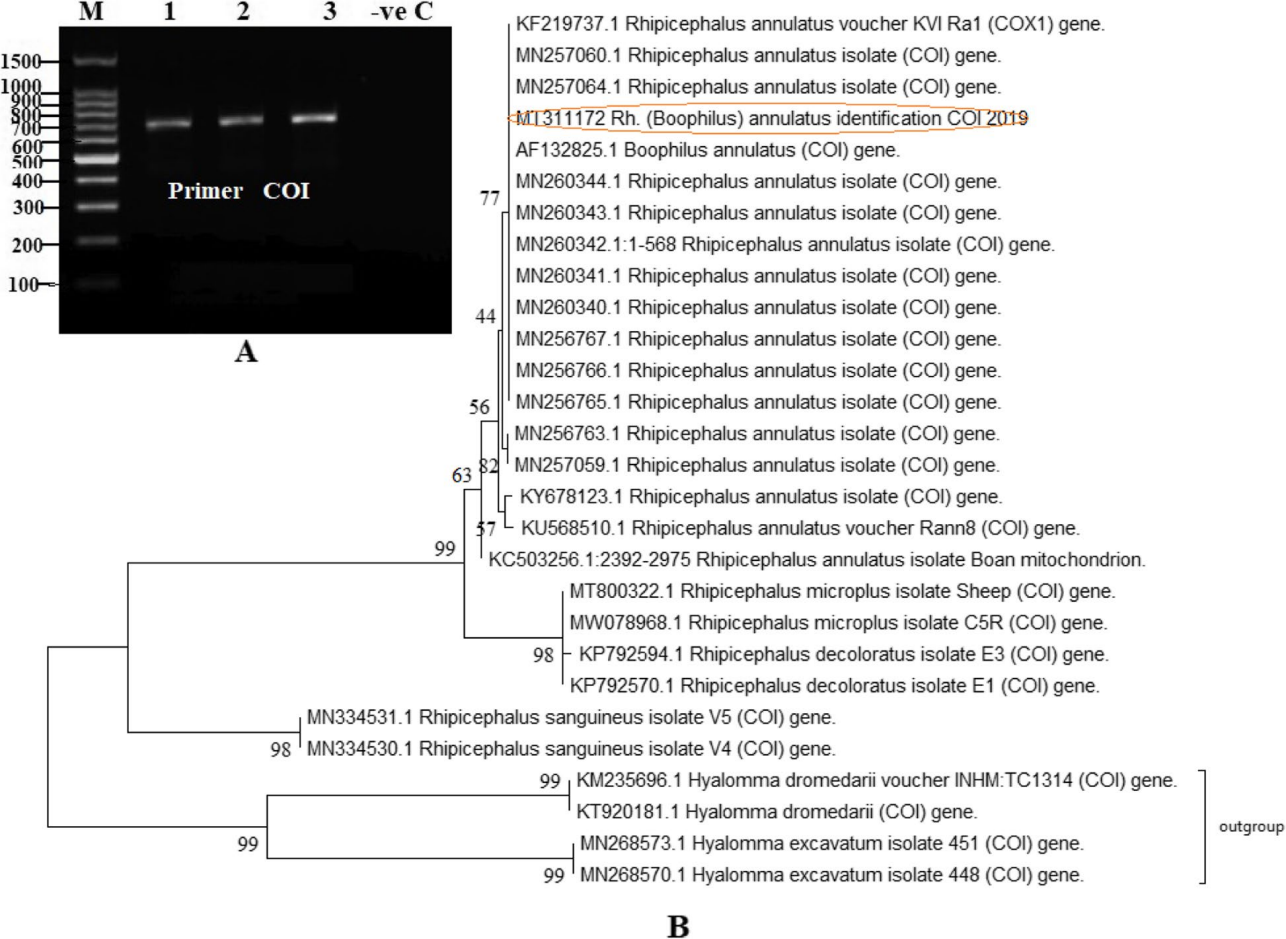
**A** Amplification of COI gene produces 678 bp of PCR products from tick species. Lane M: 100 bp DNA ladder; Lane 1: DNA of field hard ticks, *R*. (*Boophilus*) *annulatus,* collected from Al-Behera; Lane 2, 3: DNA of field hard ticks, *R.* (*Boophilus*) *annulatus,* collected from Kafr El-Sheikh and Lane –ve C: Negative control, **B** Phylogenetic tree of *R. (Boophilus) annulatus* ticks in Egypt, inferred using the Maximum Composite Likelihood method on MEGA X software. The percentage of replicate trees in which the associated taxa clustered together in the bootstrap test (1000 replicates) is shown next to the branches. *H. dromedarii* and *H. excavatum* sequence was used as outgroup

A total of 4000 adult ticks were collected from 114 cows (females) and they were morphologically identified as belonging to *R.* (*Boophilus*) *annulatus*. These findings indicated that *R.* (*Boophilus*) *annulatus* is the most prevalent tick species on cattle in the specified region in Egypt. Ticks were divided into twenty pools, each pool containing 200 ticks from various locations in the same governorate, from different Egyptian provinces. Three pools of the infected ticks out of 20 were found positive to LSDV by PCR and sequencing. This finding revealed that 15% of the whole specimens were positively infected as 600 out of 4000 have been confirmed positive to LSDV. The COI DNA gene sequences isolated from three positive pools of *R. (Boophilus) annulatus* resulted in a 678 bp amplicon (Fig. 1A) that was sequenced and deposited in GenBank under accession number MT311172. It displayed 98.4–100% similarities to the *R.* (*Boophilus*) *annulatus* sequence retrieved from the GenBank database (accession number MN260340.1 and AF123825.1). All the sequences from this study aligned with the *R. (Boophilus) annulatus* sequences from GenBank and significantly diverged from *R. microplus* sequences (Fig. 1B).

### Molecular identification of a recent local LSDV from cattle ticks collected from different governorates in Egypt

**Fig. 2:**
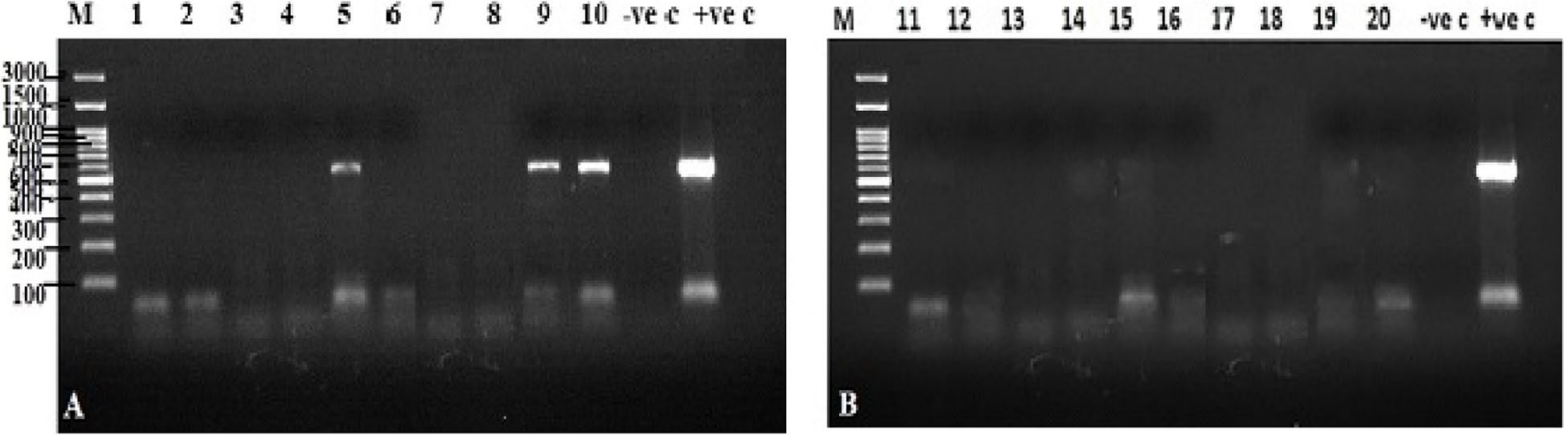
Amplified products (554 bp in size) for a local LSDV strain from tick samples using unique primers for the GPCR gene. Lane M: High molecular weight nucleic acid marker (100 bp), Tick samples collected from different governorates in 2018 and 2019 as follows, **A** Lane 1, 2: Beni Suef, Lane 3, 4: Menofya, Lane 5, 6: Al-Behera, Lane 7, 8: Sharqiya, lane 9, 10: Kafr El-Sheikh. Lane 11: Negative control, Lane 12: Positive control for LSDV, **B** Lane 11, 12: Demiatta, Lane 13, 14: ElQalyubia, Lane 15, 16: Ismailya, Lane 17, 18: Menia, Lane 19, 20: Giza, Lane 21: Negative control, Lane 22: Positive control for LSDV

Three pools (200 ticks per pool) of the screened samples tested positive for LSDV (one sample from Al-Behera governorate in the 2018 survey and two samples from Kafr El-Sheikh governorate in the 2018 and 2019 surveys) as determined by gel electrophoresis. None of the negative controls produced an amplicon (Fig. 2A and B).

### Isolation of LSDV from tick samples and inoculation into SPF-ECE

**Fig. 3:**
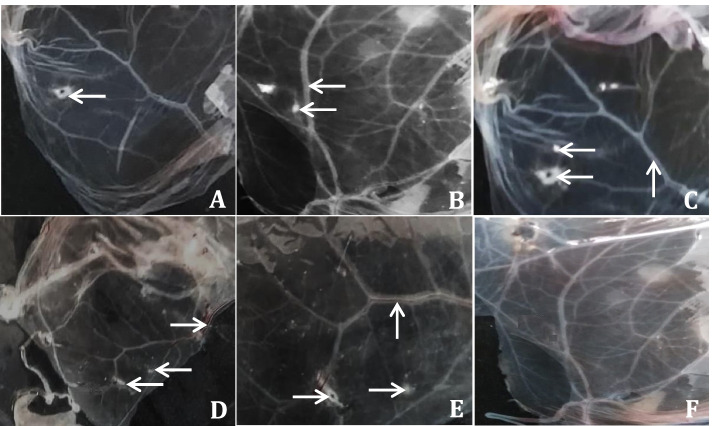
Chorioallantoic membrane of SPF-EGEs after three passages, **A, B, C, D** and **E**: characteristic pock lesion and a hemorrhagic membrane with congestion with LSDV. **F**: depicting the negative control with chorioallantoic membrane showing no inflammatory signs or pock lesions. The white arrows refer to lesion

Three pools (200 ticks per pool) of collected adult ticks from clinically suspected LSDV-infected cattle gave positive result in GPCR et al.-Behera and Kafr El-Sheikh governorates, Egypt. They were inoculated into SPF-ECE via CAM route. By the 3^rd^ passage, three pooled samples were found to be positive out of 20 pooled samples. On inspection of the collected chorioallantoic membranes, a hemorrhagic membrane with congestion, clotted blood in blood vessels, and pock lesions in the form of stretched white lines were observed. The pock lesions became more prominent after 6 days of inoculation at the 3^rd^ passage. As illustrated in, the dead embryos were hemorrhagic and edematous, with an enlarged and bloody liver and clots of blood within the core (Fig. 3).

### Transmission electron microscopy

**Fig. 4:**
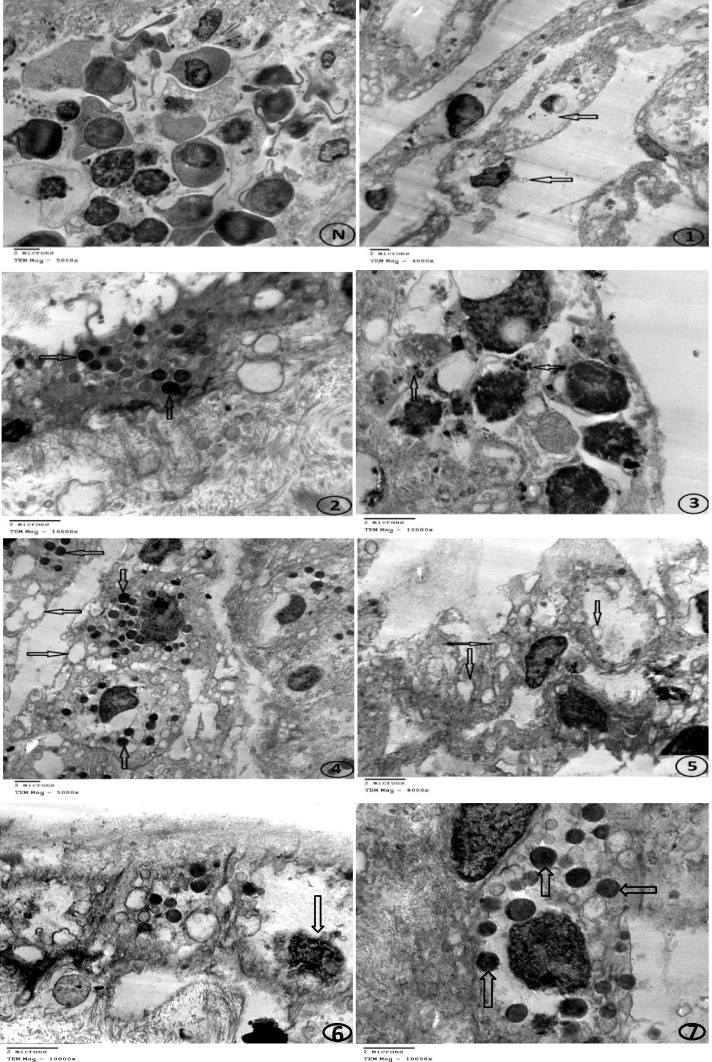
Electron micrographs of inoculated CAM with suspected LSD viral sample stained with uranyl acetate and lead citrate. N: Negative control, the arrows refer to: 1: Rupture of cells, 2 and 3: Dark inclusion bodies, 4 and 5: Vacuoles in cells, 6: Empty cells with distorted nucleus and 7: The virus particles appeared as ovoid in shape, with rounded ends

Few virus particles were observed in the infected cells and they appeared ovoid in form with rounded ends, according to EM analysis of inoculated CAM with suspected LSD viral samples. Viral protein aggregates were abundant in the cell cytoplasm as inclusions. The characteristic virus particle was released from the cellular membrane by budding as shown in Fig. 4.

### Sequencing and phylogenetic analysis of partial GPCR gene

Purified PCR products of the LSDV detected in this study were sequenced and deposited in Genbank under the accession numbers MN879402, MN879403, and MN845071. The sequence analysis of the 497 bp GPCR gene (*n* = 3) identified in this study showed that all sequences from Al-Behera and Kafr El-Sheikh, as well as tissues adapted to the MK765544.1 LSDV Kazakhstan/2016 and MN381843.1 LSDV/Egy/2015 (GPCR) genes, were identical. LSDV isolates were genetically closest and had high sequence homology, with an overall nucleotide identity of 98.9%–100%. All LSDV isolates from ticks collected in Egypt for this study were 100% identical.

**Fig. 5:**
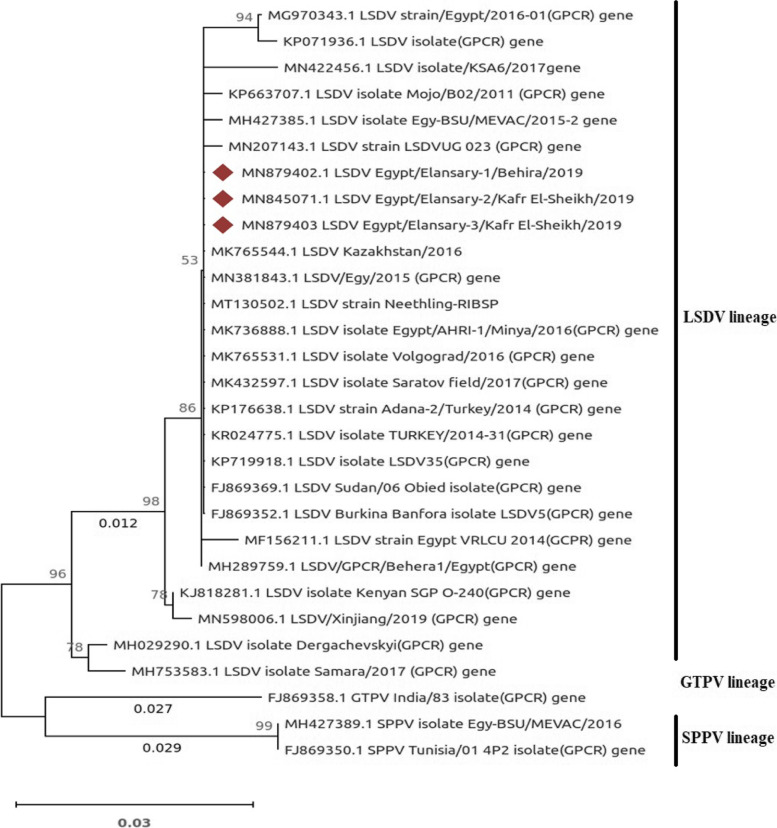
Phylogenetic analysis of detected 29 LSDV GPCR partial gene sequences including three of the sequences obtained in the current study with other GPCR published in GenBank database. The numbers below the branches represent the genetic distance between the associated taxa. The numbers above the branches represent the percentage of the 1000 bootstrap replicates in which the associated taxa clustered together. Sequences obtained in this study are indicated by black diamond shape. GTPV and SPPV sequence were used as outgroups

The LSDVs were clustered within two distinct groups, the first one is composed of vaccine viruses and the second one of field isolates. Sequences obtained from this study were clustered together with other LSDV isolates retrieved from the GenBank database. The SPPV and GTPV strains formed distinct clusters from the LSDV cluster as out groups (Fig. 5). Phylogeny on this gene reported here confirms that the CaPVs can be divided into three distinct lineages, SPPV, GTPV and LSDV where all of the sequences recorded in this study clustered together in one lineage with other LSDV sequences from cattle obtained from GenBank, including those from Egypt, Africa, and the Middle East.

## Discussion

The current study focused on the isolation of LSDV from ticks collected from cattle in the aforementioned governorates in Egypt and their inoculation into the CAM of SPF-ECE with further identification by non-serological methods, such as TEM and molecular characterization of virus isolates using PCR and sequencing, as well as the establishment of a phylogenetic tree between local LSDV strains from various sources, sheep pox and goat pox viruses. For accurate species identification of the collected ticks, COI sequencing was implemented. The universal DNA primers LCO1490 and HCO2198 [25] used in this study are widely used in species identification and phylogenetic studies [26], and genetic variability of *R. (Boophilus) annulatus* [27].

A massive outbreak of suspected LSDV-infected cattle was observed between May to November over two consecutive years, namely 2018 and 2019, in Egypt's Al-Behera, Menofya, Sharqiya, Beni Suef, and Kafr El-Sheikh governorates. It is worth noting that the majority of recent LSD outbreaks occurred during the year's mild, wet weather from May to September, which favored tick activity. LSD incidence decreased during the cold season and increased during the rainy season, which corresponds to previous studies [28]. In Egypt's 2018 LSD outbreak, the disease presented a robust clinical image, with a high mortality rate and significant economic losses in meat and milk. Male cattle showed sterility, beside abortion and the females presented infertility consistent with previous records [29]. In the present study which was conducted in Egypt's Al-Behera, Menofya, Al Sharqiya, Beni Suef, and Kafr El-Sheikh governorates between May to November over two consecutive years, the results of the isolation of LSDV showed characteristic pock lesions on the CAM of SPF-ECEs appearing as pock lesions with distributed foci typical of LSD which are in agreement with the findings of El-Tholoth and El-Kenawy [30].

The PCR results obtained from current study at the right positions for the LSDV gene are consistent with those mentioned by Allam et al*.* [31]. Furthermore, our findings were consistent with previous studies concerning isolated LSDV from skin biopsies on CAM and MDBK cell culture [13, 32].

GPCR specific for LSDV identification was used to test 20 pools of tick samples, and three of the pools were found to be positive for the virus (two in the 2018 and one in the 2019 surveys). In order to understand the epidemiology of the recent LSDV outbreaks in Egypt, this study used GPCR gene-based phylogenetic analysis for molecular identification and phylogenetic analysis of LSDV in Egyptian governorates. The additional methods are validating the results obtained by molecular characterization which is in line with previous studies [33] and help in the differentiation between LSD and pseudo-LSD caused by bovine herpesvirus 2.

Moreover, the current findings are consistent with those of El-kady et al. [32]. The viral gene detected in samples collected from the governorates of Al-Behera and Kafr El-Sheikh was identified and confirmed by conventional PCR. These findings support previous research to detect LSDV in cattle and water buffaloes in Egypt [34], in skin lesion [13, 15], and in blood and skin samples [35].

## Conclusion

The current study confirmed that the LSDV GPCR gene was found in the pooled clinical cases of Al-Behera (one sample in the 2018 survey) and Kafr El-Sheikh (two samples in the 2018 and 2019 surveys, one in each year) governorates, confirming the presence of LSDV in this outbreak. After sequencing, the LSDV gene was found to be similar to those previously detected and registered in GenBank. The positive GPCR isolation of LSDV resulted in characteristic pock lesions on the CAM of SPF-ECEs, which emerged as pock lesions of scattered foci typical of LSD and were validated with TEM. Minor differences were noticed between the nucleotide sequence obtained in the current study and other previous isolates registered on Genbank indicating the genetic drift which is a normal phenomenon.

## Recommendations

It is recommended to regularly make surveys to field strain of LSDV in Egypt because of the influence of genetic drift that may induce changes in the sequences of temporally different isolates. Interestingly, effective vaccines should be established to prevent future outbreaks.

## Methods

### Ethical statements

All experiments were performed in accordance with relevant guidelines and regulations. The study has been approved by the Ethics Committee of the Faculty of Science, Al-Azhar University, Egypt. All methods are reported in accordance with ARRIVE guidelines [36]. We have obtained informed permission/consent from the cattle owners before collection of sample/ticks.

### Animal selection, tick collection, and identification

**Fig. 6:**
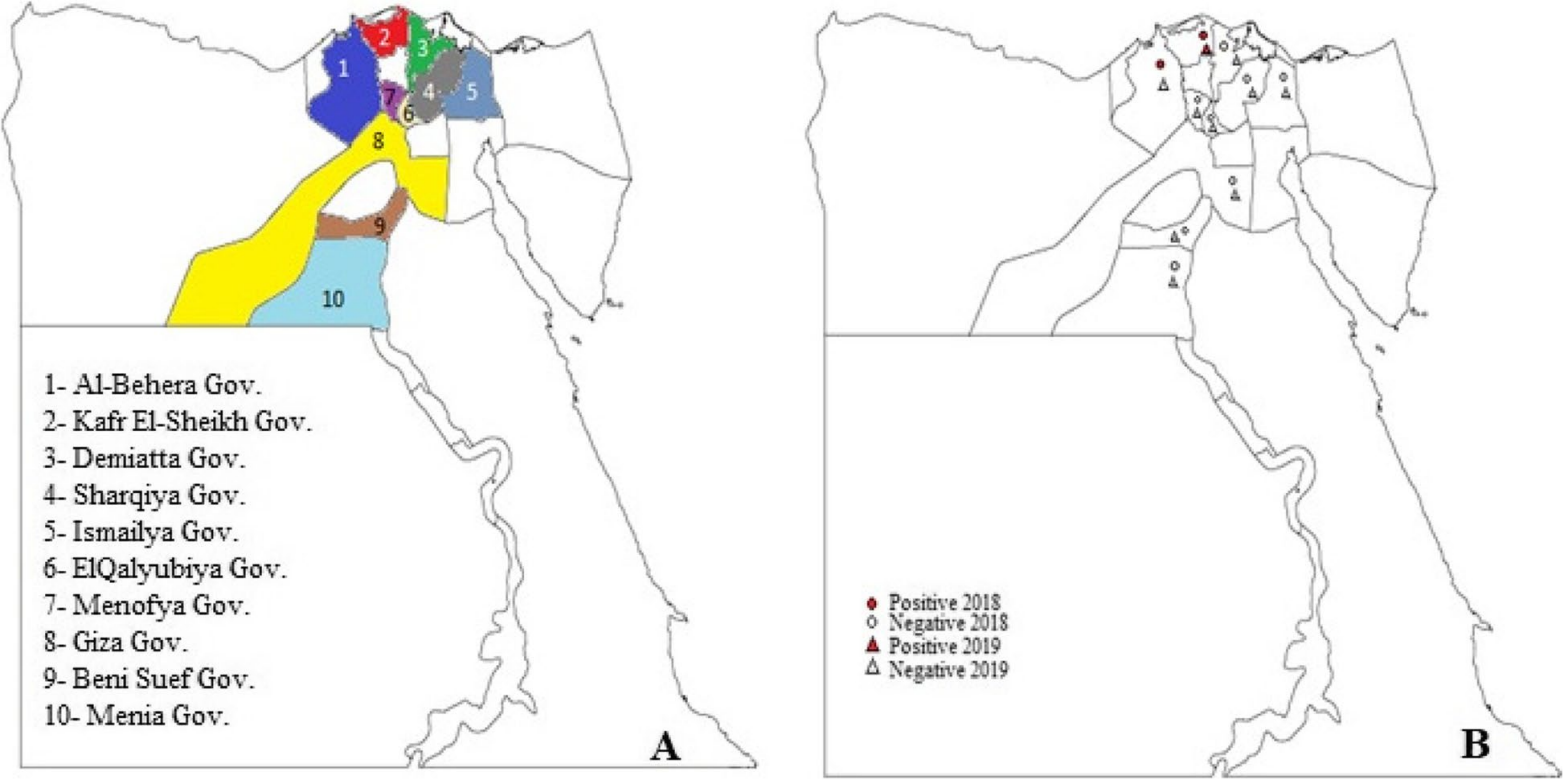
Map illustrated the geographical distribution of collected tick samples in this study from different localities in Egypt, **A**: Egyptian governorates, and **B**: Positive or negative samples of LSDV

Cattle farms under investigation were chosen based on the presence of clinical signs consistent with LSD between May to November over two consecutive years, namely 2018 and 2019, in the Egyptian provinces of Sharqiya, Menofya, Al-Behera, Kafr El-Sheikh, Qalyubia, Demiatta, Ismailya, Menia, Beni Suef, and Giza. These farms were visited, and all suspected cases were clinically examined, and the hard tick *R. annulatus (formerly Boophilus*) was collected from the cows with suspected LSDV infection. Live ticks were brought to the Animal Health Research Institute in Dokki, Egypt. A total of 4000 adult ticks were collected from 114 cows (females), divided into twenty pools (each pool containing 200 ticks from various locations in the same governorate) from different Egyptian provinces (Fig. 6). Ticks were collected from various body parts of cows and kept at -70 °C until used. They were morphologically identified using taxonomic keys [37] and molecularly identified using the COI gene in the laboratory of the Zoology and Entomology department, Faculty of Science, Al-Azhar University, Cairo, Egypt.

### Preparation of field ticks for detection of LSDV and virus isolation

Collected tick samples were washed twice with sterile water to remove excess particulate contamination, rinsed once with 70% ethanol, cut into small pieces, and ground with phosphate buffered saline (PBS) using a sterile mortar and pestle. Two samples were used to represent each province, with each sample containing approximately 200 ticks. At 4 °C, each tissue homogenate was centrifuged for 10 min at 3000 rpm (1008 xg) and the clear supernatant was frozen at -70 °C for PCR and virus isolation.

### Molecular identification

#### DNA extraction, gene amplification, and sequencing

Identification of LSDV and *R. (Boophilus) annulatus* in Egypt was confirmed using GPCR gene sequences and COI gene sequences, respectively. First, GeneJET Genomic DNA Purification Kit (Thermo Scientific, Cat. no. K0721) was used to extract DNA according to the manufacturer's instructions. To confirm LSDV diagnosis using conventional PCR, twenty pools of tick samples were used. The identification was performed using LSDV forward primer 5'-AGT ACA GTT AGT AGC GCA ACC-3', and reverse primer 5'-GGG TGA ACT ACA GCT AGG TAT C-3’for virus [38], and using LCO1490 forward primer 5'-GGT CAA CAA ATC ATA AAG ATA TTG G-3', and reverse primer 5'-TAA ACT TCA GGG TGA CCA AAA AAT CA-3' for tick [39]. As a positive control, the LSDV18 Ismailya 89 Egyptian strain (accession number FJ869377) was used [7]. The PCR reaction mixture was adjusted to 25 µL containing 5 µL of DNA, 12.5 µL of AmpliTaq Gold® 360 Master Mix, 3 µL of each primer, and 1.5 µL of nuclease free water as per the kit manual instructions. Amplification of the target genes was carried out in a BIO-RAD® PCR system T100 thermocycler (BioRad, Hercules, California, USA) as previously described [40]. For LSDV, an initial step of 95 °C for 10 min (predenaturation) was followed by 40 cycles of 95 °C for 30 s (denaturation), 50 °C for 30 s (annealing), and 72 °C for 1 min (extension) in the PCR program. While for tick identification, the temperature profile was 95 °C for 5 min (predenaturation) followed by 35 repeated cycles, each for 1 min of 95 °C (denaturation), 42 °C (annealing), and 72 °C (extension). In both cases, the program is then set to 72 °C for 10 min (post extension) and scheduled for a final hold at 4 °C.

The 554 bp DNA amplicon was visualized and detected for LSDV using the Molecular Imager Gel Doc™ XR + Imaging system (BIO-RAD) and Image lab™ software for gel image analysis. According to the manufacturer's instructions, positive PCR-products were purified using the QIAquick® Gel Extraction Kit (QIAGEN, USA, Cat. no. 28704) and then sequenced with the same previous primers using BigDye® Terminator v3 and cycle sequencing kit.

## Sequence alignment and phylogenetic analyses_sec3

BLAST similarity search option (available at http://www.ncbi.nlm.nih.gov) was used to search the GenBank database for tick identification. Additionally, sequences containing SPPV and GTPV for LSDV and sequences containing *Hyalomma dromedarii* and *Hyalomma excavatum* for *R. (Boophilus) annulatus* were included to determine evolutionary relationships. Phylogenetic tree was constructed using Maximum Likelihood and the Tamura-Nei model [41] by MEGAX inferred from 1000 bootstrap replicates [42]. Resulted sequences were deposited in GenBank database.

### Virus isolation in CAM

Tick samples were homogenized in PBS (pH 7.4) containing 100 units of penicillin and 100 mg of streptomycin. The homogenate was lysed three times by freezing and thawing, and the supernatant was purified by centrifugation at 3000 rpm for 15 min at 4 °C, filtration through a 0.45 µm pore-size cellulose acetate filter, and incubation at room temperature for one hour. SPF-ECEs (4 for each sample) were examined (external and internal) and incubated in an egg incubator at 37 °C for 9—11 days with daily examination by an egg candler, regular shaking, ventilation, and controlled humidity. CAM was inoculated with 0.2 ml of the supernatant and samples were passaged three times in ECE. The eggs were examined for 5–7 days after inoculation. Nonspecific death was assigned to embryos that died within the first 24 h. Pathoanatomic signs were recorded on chick embryos and CAM.

### Identification of a suspected LSD virus isolate by transmission electron microscopy

As described previously, a thin section of infected CAM was prepared for EM [35]. Where the CAM was fixed in 4% buffered glutaraldehyde, post-fixed in 1% buffered osmium tetroxide, dehydrated in a graded series of ethanol solutions, and finally embedded in an epoxy resin. Reichert-Jung Ultra-cut 701,701 UCT Ultramicrotome sections were cut semi-thin, stained with toluidine blue, and examined under a light microscope. The ultrathin sections were mounted on copper hexagonal grids, stained with uranyl acetate and lead citrate [43], and examined using a TEM (JEM1010-JOEL-Japan) at the Regional Center for Mycology and Biotechnology (RCMB), Al-Azhar University, Cairo, Egypt.

## Data Availability

All data generated and analyzed in this study are included in this article. The nucleotide sequence data that support the findings of this study are openly available in the GenBank database at http:// www.ncbi.nlm.nih.gov/genbank/, accession numbers MN879402, MN879403, and MN845071.
